# Selectivity and Resolving Power of Hydrophobic Interaction Chromatography Targeting the Separation of Monoclonal Antibody Variants

**DOI:** 10.1021/acs.analchem.3c04011

**Published:** 2024-01-08

**Authors:** Raphael Ewonde Ewonde, Katharina Böttinger, Jelle De Vos, Nico Lingg, Alois Jungbauer, Christopher A. Pohl, Christian G. Huber, Gert Desmet, Sebastiaan Eeltink

**Affiliations:** † Department of Chemical Engineering, Vrije Universiteit Brussel (VUB), Pleinlaan 2, 1050 Brussels, Belgium; ‡ Department of Biosciences and Medical Biology, Bioanalytical Research Laboratories, University of Salzburg, Hellbrunner Strasse 34, 5020 Salzburg, Austria; § Department of Biotechnology, Institute of Bioprocess Science and Engineering, University of Natural Resources and Life Sciences, Muthgasse 18, 1190 Vienna, Austria; ∥ CAP Chromatography Consulting, Sunnyvale, California 94587, United States

## Abstract

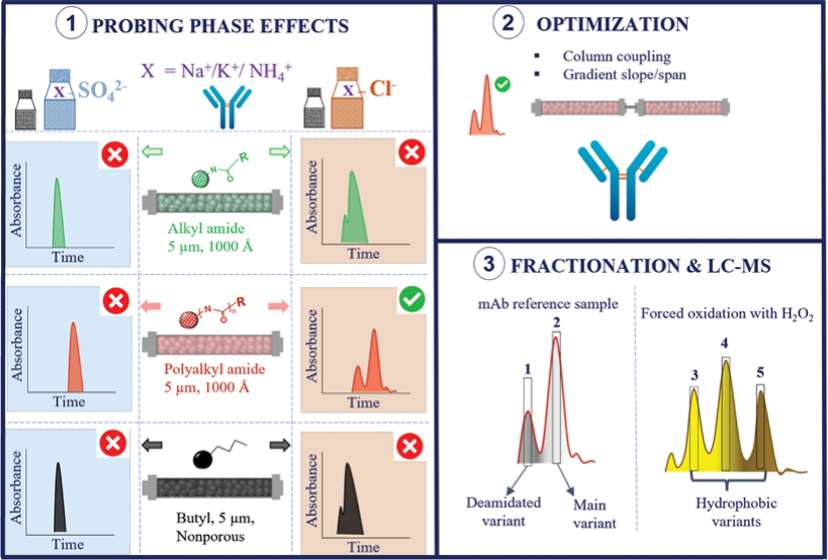

This study presents a comprehensive investigation of the mechanistic understanding of retention and selectivity in hydrophobic interaction chromatography. It provides valuable insights into crucial method-development parameters involved in achieving chromatographic resolution for profiling molecular variants of trastuzumab. Retention characteristics have been assessed for three column chemistries, i.e., butyl, alkylamide, and long-stranded multialkylamide ligands, while distinguishing column hydrophobicity and surface area. Salt type and specifically chloride ions proved to be the key driver for improving chromatographic selectivity, and this was attributed to the spatial distribution of ions at the protein surface, which is ion-specific. The effect was notably more pronounced on the multialkylamide column, as proteins intercalated between the multiamide polymer strands, enabling steric effects. Column coupling proved to be an effective approach for maximizing resolution between molecular variants present in the trastuzumab reference sample and trastuzumab variants induced by forced oxidation. Liquid chromatography–mass spectrometry (LC–MS)/MS peptide mapping experiments after fraction collection indicate that the presence of chloride in the mobile phase enables the selectivity of site-specific deamidation (*N*_30_) situated at the heavy chain. Moreover, site-specific oxidation of peptides (*M*_255_, *W*_420_, and *M*_431_) was observed for peptides situated at the Fc region close to the CH2–CH3 interface, previously reported to activate unfolding of trastuzumab, increasing the accessible surface area and hence resulting in an increase in chromatographic retention.

## Introduction

Over the past two decades, therapeutic proteins and peptides have emerged as the major products in the biopharmaceutical industry pipeline. Among them, monoclonal antibodies (mAbs) are the primary biopharmaceutical product due to their high target specificity and safe toxicology profile.^1^ However, unlike small-molecule drugs, which are produced by controlled chemical reaction schemes, mAbs are predominantly produced using mammalian cell lines.^2^ This invariably leads to post- and cotranslational modifications, which can be both enzymatic (such as glycosylation)^3,4^ and chemical (including oxidation, deamidation, isomerization, etc.),^5−7^ resulting in increased heterogeneity that potentially affects their biological activity.^8^ Regulatory agencies have imposed stringent directives for impurity profiling of drug substances that can arise during the manufacturing process and/or storage.^7^ As such, chromatographic techniques play a crucial role in protein characterization, as they enable the identification and quantification of impurities and modifications present in protein samples.^9^

Hydrophobic interaction chromatography (HIC) offers the possibility of working under nondenaturing conditions, and it is a widely used chromatographic technique that finds application at different stages of downstream processing.^10,11^ HIC has also emerged as an analytical separation technique for profiling biologics.^12,13^ The current state-of-the-art stationary phases for analytical HIC separations are primarily based on particulate materials functionalized with alkyl or alkylamide moieties that yield a weakly hydrophobic surface chemistry.^14^ A conventional HIC method involves the application of an inverse salt gradient, typically ammonium sulfate. Examples of analytical HIC applied to the analysis of modifications in recombinant mAbs include the work of Valliere-Douglass et al.^15^ They demonstrated the potential of HIC for the separation of antibody variants, resulting from variable N- and C-terminal processing, stress-induced modifications, and conformationally altered populations present in the drug product. Similarly, an HIC method for monitoring light-chain succinimide and isomerization variants was described.^16^ Boyd et al. reported on HIC IgG1 profiling after forced oxidation and showed that IgG1 with an oxidized tryptophan at the complementarity determining region (CDR) eluted prior to the main IgG1 peak.^17^ Modifications in the complementarity determining region (CDR) generally result in changed specificity of the mAb and are usually a critical quality attribute.

Although significant progress has been made in the development of HIC methods, there are still some knowledge gaps that need to be addressed, particularly in the profiling of monoclonal antibody variants. These gaps pertain to our understanding of how both stationary phase particle architecture and chemistry, as well as the chemical composition of the salt system (including ions), impart retention and selectivity for achieving the best resolving power. Further, identification of the optimal gradient elution conditions remains a critical aspect that requires further investigation. This study aims to comprehensively assess HIC retention and selectivity applied to mAb variants and impurity profiling. To this end, the IgG1 antibody trastuzumab has been analyzed as a reference sample, and variants were artificially generated through forced oxidation. To elucidate critical method parameters, stationary phase surface area and ligand chemistry, mobile-phase salt composition and concentration, column length, and gradient steepness were systematically varied and the effects on chromatographic retention, selectivity, and resolution were evaluated. Finally, reverse phase high-performance liquid chromatography-mass spectrometry (RP-HPLC-MS/MS) analysis at the peptide level was performed on isolated HIC fractions to study mAb microheterogeneity and to gain insights on retention order of trastuzumab variants in HIC.

## Experimental Section

### Chemicals and Reagents

Ammonium sulfate (≥99.0%), sodium chloride (≥99.5%), sodium sulfate (≥99%), potassium chloride (≥99%), disodium hydrogen phosphate (≥99.0%), sodium dihydrogen phosphate (≥99.0%), hydrogen peroxide solution (30% w/w), sodium hydroxide solution (HPLC grade, 50.0%), and formic acid (>98%) were purchased from Sigma-Aldrich (Bornem, Belgium). Acetonitrile (ACN, HPLC-MS grade) was purchased from VWR International (Shanghai, China), and trypsin (porcine) was obtained from Promega (Mannheim, Germany). Ultrapure water was produced in-house using a water purification system (Milli-Q Integral 3, Merck/Millipore, Billerica).

### IgG Production and Forced Oxidation of IgG

Anti-Her2 IgG1 was produced in a Chinese hamster ovary (CHO) fed-batch cell culture according to Sissolak et al.^18^ and subsequently purified using a Toyopearl AF rProtein A HC-650F affinity material (Tosoh, Stuttgart, Germany), yielding the trastuzumab reference material. 96 μL of 12 mg/mL mAb was mixed with 4 μL of 30% (w/w) hydrogen peroxide and incubated for 24 h at 6 °C to induce protein oxidation. Next, the sample was transferred to an Amicon Ultra (10 kDa MWC) spin vial, 400 μL of Milli-Q water was added, and the sample was centrifuged for 5 min at a maximum speed using a Micro Star12 centrifuge (Avantor, Leuven, Belgium). Buffer exchange was performed with 20 mM Tris-HCl buffer, pH 7.1, and ∼80 μL of the supernatant was collected.

### HIC Instrumentation and Conditions

HIC experiments were conducted using an Ultimate 3000 bioinert HPLC system (Thermo Fisher Scientific, Germering, Germany) composed of a low-pressure gradient pump, a well-plate autosampler enabling inline split-loop injections, a forced-air column oven, and a diode-array UV detector. Chromeleon software (version 7.2.10) was used for system control and data management. The injection volume was set to 5 μL. The temperature of the well-plate sampler was set at 6 °C and the column oven was maintained at 30 °C. A 2.5 μL UV flow cell was used at 210 and 280 nm with a data collection rate of 5 Hz and a response time of 1 s. The columns used in this study were 4.6 mm I.D. × 100 mm packed with 5 μm porous (1000 Å) silica particles functionalized with alkylamide chemistries (MabPac HIC-10 and MabPac HIC-20) or packed with 5 μm nonporous polymer particles functionalized with butyl chemistry (HIC-butyl) obtained from Thermo Fisher Scientific (Sunnyvale). HIC analyses were generally performed in gradient mode by applying inverse salt gradients starting at 2 M (NH_4_)_2_SO_4_ or 4 M NaCl in 50 mM phosphate buffer and decreasing the salt concentration linearly in time. All mobile phases were filtered through a 0.22 μm filter (Millipore, Darmstadt, Germany).

### RP-HPLC-MS/MS Peptide Profiling

HPLC-MS/MS experiments were performed using an Ultimate 3000 RSLCnano coupled via nano-ESI (NSI) to a Q Exactive Hybrid Quadrupole-Orbitrap mass spectrometer (Thermo Fisher Scientific). Proteins were denatured with 5 mM tris(2-carboxyethyl)phosphine (Sigma-Aldrich) at 60 °C and 900 rpm for 30 min. Disulfides were alkylated using 20 mM iodoacetamide (Sigma-Aldrich) at 22 °C and 750 rpm in darkness for 45 min. Protein subunits were purified using C_18_ pipet tips (Thermo Fisher Scientific), dried under vacuum (ScanVac, Lynge, Denmark), and digested with trypsin (Promega, enzyme/protein ratio = 1:25) overnight at 37 °C.

Resulting peptides were separated using a 75 μm I.D. × 150 mm PepMap Neo column packed with 2 μm C_18_ particles with 100 Å pores (Thermo Fisher Scientific) with 0.1% FA in H_2_O (A) and ACN (B) applying the following gradients: 1% B for 5 min, 1–30% B in 40 min, 30–60% B in 10 min, 99% B for 5 min, and 1% B for 20 min at 0.3 μL/min and 50 °C. The spray voltage for the NSI source probe was set at +1.4 kV, a probe heater temperature and capillary temperature at 350 and 250 °C, respectively, and an S-lens RF level at 60. Peptides were analyzed in a data-dependent MS/MS mode. Full scan was operated within a scan range of *m*/*z* 400–3000 at a resolution setting of 70,000, with an AGC target of 3e6, and a maximum injection time of 150 ms. Ten data-dependent scans were acquired at a normalized collision energy of 28 with a resolution setting of 17,500, an AGC target of 1e5, a maximum injection time of 150 ms, and a dynamic exclusion of 10 s. MS data were analyzed using Byonic (v. 3.11.3, Protein Metrics Inc., Cupertino). Peptides were quantified using Skyline (v. 20.2).

## Results and Discussion

### Retention and Selectivity Assessment

The retention characteristics of a mAb was evaluated on three HIC column chemistries, i.e., a conventional HIC phase based on butyl ligands (HIC-butyl), a bonded phase based on short strands of polymeric alkylamide, and a bonded phase based on long strands of a multialkylamide ligand. Table 1 provides an overview of stationary phase properties.

**Table 1:**
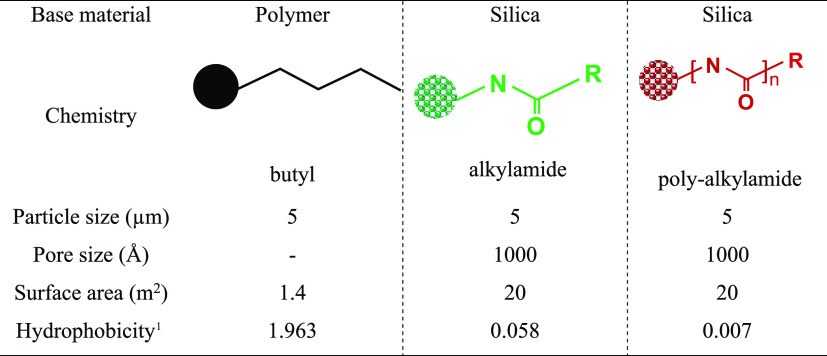
Stationary Phase Characteristics

1 Determined using phenanthrene as the tracer in isocratic mode, applying 30:70 (v/v)%, ACN:0.1 M NH_4_CH_3_COOH, pH 5.4, as the mobile phase.

**Figure 1:**
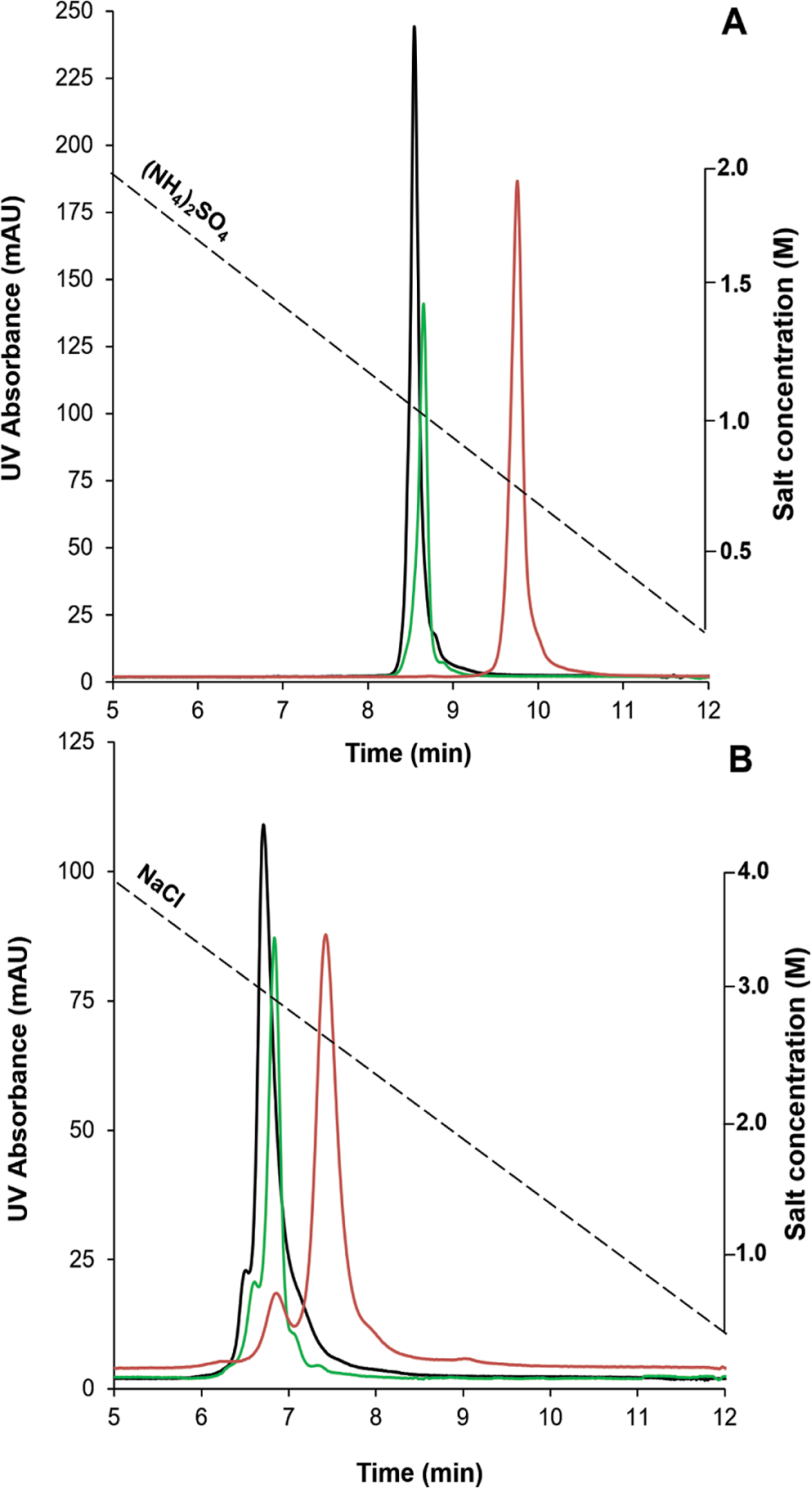
Comparison of retention and selectivity obtained on different HIC columns for unstressed trastuzumab, applying inverse gradients of NH_4_(SO_4_)_2_ from 2 to 0 M (A) and NaCl from 4 to 0 M (B) on a butyl phase (black profile), a bonded phase based on short strands of polymeric amide (green profile), and a covalently attached stationary phase based on a multiamide ligand (red profile). *F* = 1 mL/min, *t*_G_ = 10 min, and the column oven was maintained at 30 °C.

Figure 1 shows the peak profiles of the trastuzumab reference sample injected on the different columns by applying ammonium sulfate (Figure 1A) and sodium chloride (Figure 1B) salt systems. To distinguish the contribution of stationary phase hydrophobicity and surface area (porous vs nonporous particles) on the resulting retention time, we first estimated the retention equilibrium constant (*K*) as an index for stationary phase hydrophobicity for the individual materials. This was done in an isocratic experiment while injecting phenanthrene as a tracer and applying:
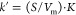
1where *k*′ is the retention factor, *S* is the surface area upon which retention is occurring, and *V*_m_ is the volume of the mobile phase. The corresponding calculations are detailed in the Supporting Information, and the resulting *K* values have been listed in Table S1. The butyl phase (Figure 1, black trace), being 33 × more hydrophobic, yielded similar retention characteristics as the short strands of polymeric amide, considering that the surface area is significantly lower (nonporous vs. porous particles). The carbonyl group in the amide phases can form hydrogen bonds with the analyte, which may also contribute to chromatographic retention. While the surface areas of the alkylamide (Figure 1, green trace) and multialkylamide (Figure 1, red trace) materials are similar and the hydrophobicity of the latter material is a factor 8 lower, the multialkylamide phase displays higher retention times. The multiamide ligand provides a mixed-mode mechanism for macromolecules, including hydrophobic, H-bonding, and dipole interactions. Moreover, as the stationary phase is highly branched, the strands project into the surrounding solution allowing macromolecules (domains) to intercalate between the polymer strands, effectively increasing the number of contact points. Antibodies are quite compact molecules due to their highly ordered structure, which is substantially maintained under HIC conditions. Although the total “cross section” of an antibody is in the range of approximately 140 × 120 Å, subdomains of the antibody, such as crystallizable fragment (Fc) or the antigen binding fragments (Fab), are considerably smaller in the range of 5–10 Å,^19^ which may be small enough at least to partially penetrate the hydrophobic surface layer of the HIC stationary phase. It should be noted that ionic interactions with any residual ionic moieties present in the alkylamide phases are suppressed by the high salt content used. When applying a less kosmotropic salt, i.e., a NaCl gradient from 4 to 0 M (Figure 1B), a decrease in overall retention time was observed but the retention order remained unchanged. This decrease in chromatographic retention is consistent with the molal surface tension increment of the salts; at equal molal concentration, salts with higher molal surface tension will yield a comparatively higher retention.^20^ Using NaCl, a separation between the main peak and the preceding shoulder became apparent, which can be attributed to oxidized variants from the main component. Hence, the salt type is a key driver for altered selectivity. The effect was most pronounced on the multialkylamide column, as proteins intercalate between the multiamide polymer strands, leading to steric effects.

**Figure 2:**
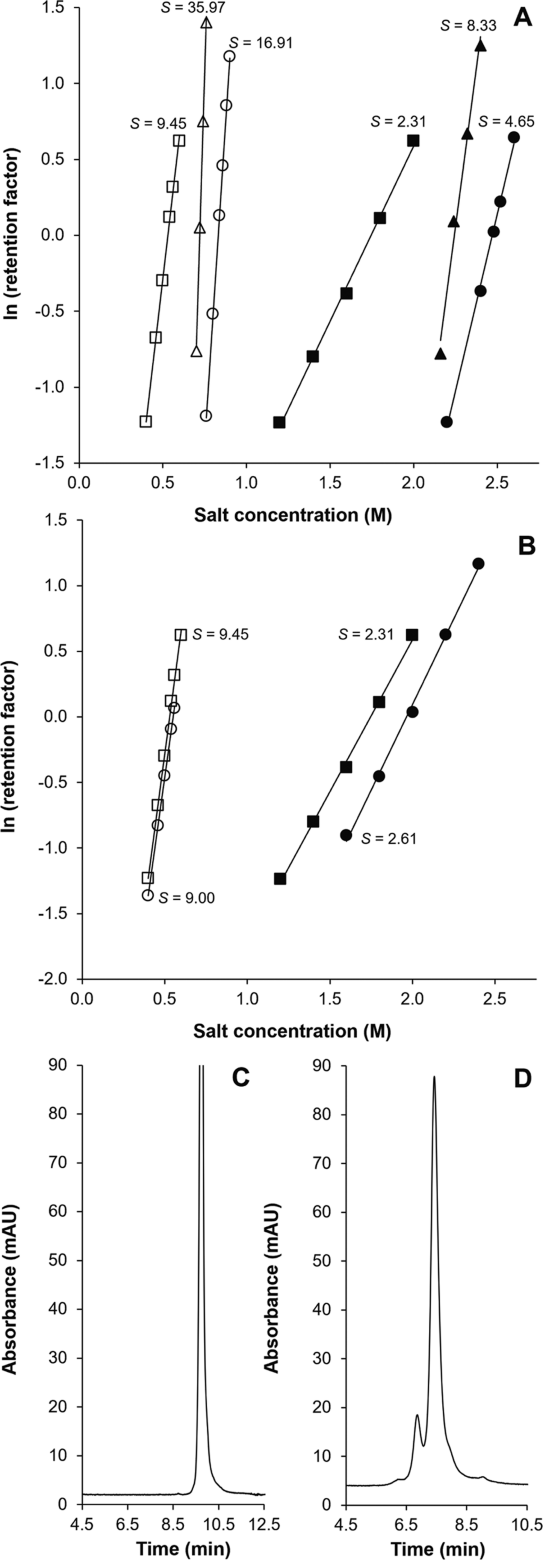
Effect of salt concentration on the retention factor of unstressed trastuzumab in the isocratic mode. (A) Chromatographic retention measured on the butyl phase (triangles), polymeric alkylamide phase (circles), and multialkylamide phase (squares) columns, applying (NH_4_)_2_SO_4_ (open symbol) and NaCl (closed symbol). (B) Retention of the trastuzumab main peak (squares) and prepeak (circles) measured on the multiamide ligand while applying (NH_4_)_2_SO_4_ (open symbols) and NaCl (closed symbols). (C) Separation of the trastuzumab reference standard using a Na_2_SO_4_ gradient and (D) separation of the trastuzumab reference standard using a KCl gradient, conditions were equal to those in Figure 1.

To obtain a more detailed understanding of the retention properties, plots of ln *k*′ vs the salt concentration were generated and analyzed (Figure 2A). The trastuzumab reference sample was injected on all three stationary phases using either (NH_4_)_2_SO_4_ (open symbols) or NaCl (closed symbols) as modifiers in isocratic mode. The linear trend lines (*R*^2^ values ≥ 0.99) indicate that the mechanism of interaction was not altered within the salt concentration range applied. Both the intercept (*y*-axis at *M*_0_) and the slope of the ln *k*′ vs [*M*] trend lines differed significantly for the different stationary phase types, see Table S2. Previously, the intercept has been correlated to the degree of hydrophobicity of the stationary phase.^21^ A notable difference in the intercept (at [*M*_0_]) was observed upon extrapolation of the retention time data between (NH_4_)_2_SO_4_ and NaCl. This observation suggests that the intercept is influenced by one or more factors, in addition to the hydrophobicity and surface area of the stationary phase. The impact of salt on retention in HIC has been attributed to the influence of molal surface tension.^22^ Previously, we observed for intact protein analysis that the retention for (NH_4_)_2_SO_4_, Na_2_SO_4_, and K_2_SO_4_ salt systems is predominantly governed by the molal surface tension increment induced by these different salts.^23^ However, here, we noticed that retention for NaCl was significantly lower than anticipated. Moreover, the slope of the ln *k*′ vs surface tension plot (for the same protein) was less steep when using NaCl. When comparing (NH_4_)_2_SO_4_ and NaCl for the analysis of trastuzumab, we observed a similar trend. This may indicate that the solvent-accessible surface area is affected.

In the next experiment, the peak profiles in the reference sample (henceforth referred to as the prepeak and main peak) were collected *via* an offline fractionation experiment, and the fractions were reinjected on the multialkylamide column, applying (NH_4_)_2_SO_4_ or NaCl as the salt systems, see Figure 2B. Within the same salt system, the lines remained virtually parallel, indicating a similar molecular contact area. However, while the trend lines of the pre- and main peak almost overlap in (NH_4_)_2_SO_4_, a vertical shift can be observed in NaCl, indicating a difference in selectivity. To track the origin of the selectivity enhancement, we performed an experiment, in which we systematically replaced the cation in the salt system. When switching from (NH_4_)_2_SO_4_ to Na_2_SO_4_, no selectivity enhancement was observed, see Figure 2C, and the chromatographic profile remained similar. Conversely, when applying KCl instead of NaCl, the enhanced selectivity with a similar chromatographic profile was maintained (see Figure 2D). Hence, Cl^–^ is a key driver for observed chromatographic selectivity. Note that an experiment with NH_4_Cl was omitted, as a very high salt concentration was required for retention, making it impractical for use. It has been previously reported that the spatial distribution of ions close to the protein surface varies for different types of cations and anions.^24,25^ We hypothesize that this effect essentially results in a difference in the exposure of hydrophobic residues at the protein surface, inducing a difference in the preferential binding area and, consequently, contributing to a difference in chromatographic selectivity as the solvent-accessible surface area changes.

### Optimizing the HIC Resolving Power

**Figure 3:**
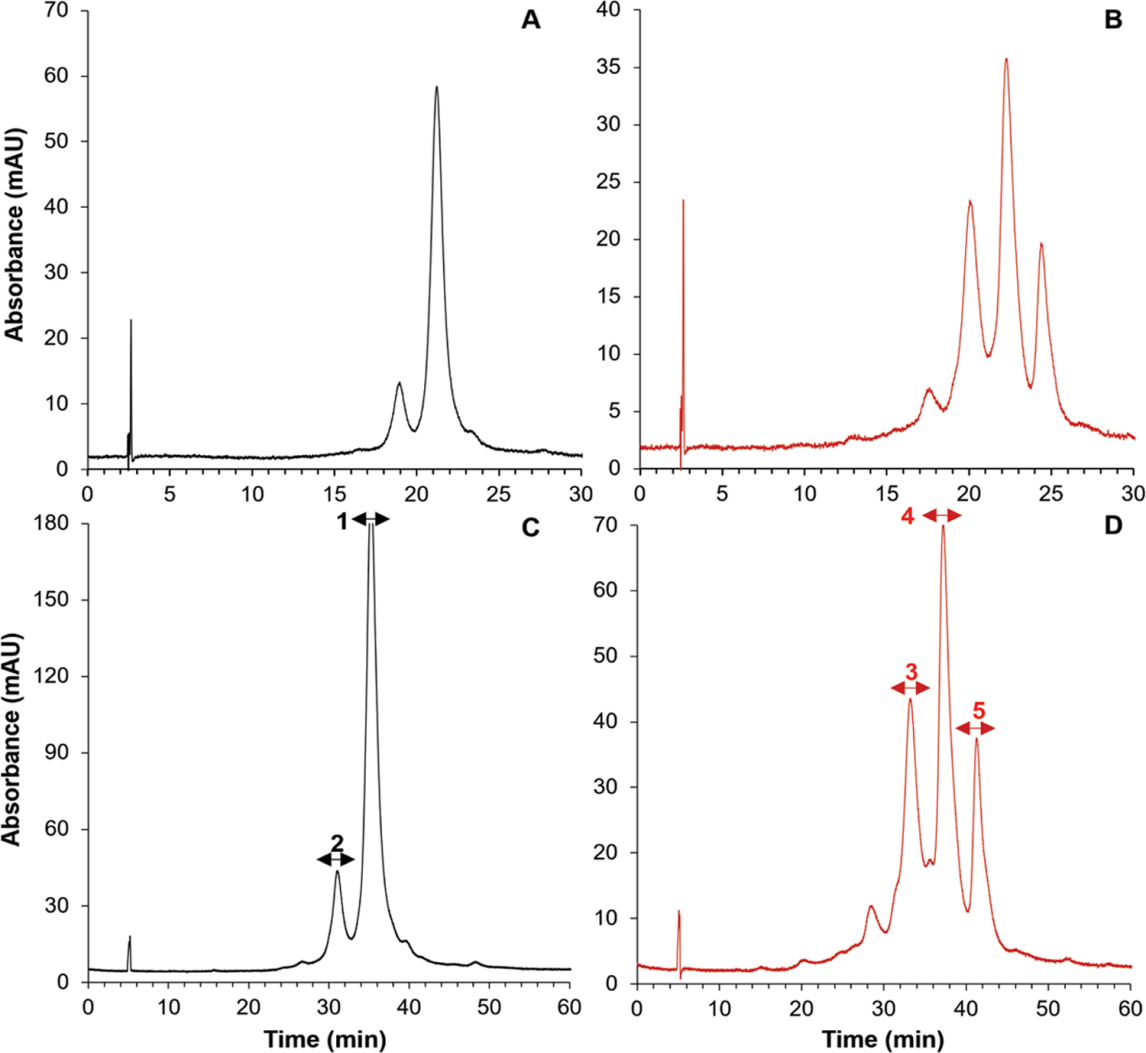
Separation of the trastuzumab reference sample and trastuzumab variants after forced oxidation on one column (A, B) and two serially coupled columns (C, D). Separations were performed using the multialkylamide stationary phase, applying an inverse gradient of NaCl from 3 to 0 M, *F* = 0.5 mL/min, *t*_G_ = 30 min (single column) and *t*_G_ = 60 min (coupled columns). The fractions that were collected for subsequent RP-HPLC-MS/MS peptide mapping experiments, highlighted by the arrows (C, D).

To generate an IgG sample with a substantial amount of oxidized amino acid residues, forced oxidation with hydrogen peroxide was pursued. To optimize the resolution between the resulting antibody variants, the effects of gradient span (Δ*c*), gradient duration (*t*_G_), and column length were systematically assessed by injecting the reference standard before and after oxidation. As the determination of the peak width (even at half height) did not result in reliable performance metric, the valley-to-peak ratio (*V*/*P*) introduced by Christophe was used to evaluate performance, where *V* is the height of the valley between the critical peak pair and *P* is the height of the lowest peak, the lower the *V*/*P*, the better the separation.^26^Figure 3A shows the separation of the reference sample (black trace) and the separation of the antibody variant generated after forced oxidation is shown in Figure 3B (red trace) obtained on a 100 mm long column after optimizing Δ*c* (3–0 M) and *t*_G_ (30 min). When applying only a narrow gradient span from 2 to 0 M, the critical pair elutes close to the column hold-up time and the gradient duration is only utilized to a very small extent, resulting in an increase in *V*/*P* and decreasing the resolving power (data not shown). In the next step, the flow rate and gradient duration were systematically varied. Applying Δ*c* of 3–0 M, a flow rate of 1 mL/min in combination with a *t*_G_ of 30 min yielded a *V*/*P* of 0.40. Increasing *t*_G_ led to a slight decrease in resolving power as the peak width increased linearly with gradient duration. On the other hand, reducing the flow rate to 0.5 mL/min led to an improved separation, yielding a *V*/*P* of 0.34. Operating at a lower flow rate results in a decrease in the mass-transfer contribution to the overall peak broadening, surpassing the impact of gradient steepness (*t*_G_/*t*_0_) on resolving power. To further optimize the separation, the effect of column coupling was assessed. Applying two coupled columns led to a further decrease in *V*/*P*, yielding 0.31, see Figure 3C (reference sample) and Figure 3D (trastuzumab after forced oxidation), respectively. Note that *t*_G_/*t*_0_ was scaled to column length, allowing an unbiased comparison with the performance obtained on one column. While one column displays limited resolution, novel oxidation variants become apparent in the coupled-column experiment.

### RP-HPLC-MS/MS Peptide Profiling of Major HIC Fractions

To get better insights into HIC retention and elution order of mAb variants, fraction collection was performed, as indicated in Figure 3C,D, prior to subsequent RP-HPLC-MS/MS analysis to determine the predominant modifications and their respective sites after protein digestion. The pre- and main peaks of the reference sample (fractions 1–2) and three main peaks present in the sample after forced oxidation (fractions 3–5) were collected. Peptide analysis targeting post-translational modifications, including *N*-glycosylation, oxidation, and deamidation, was performed.

**Figure 4:**
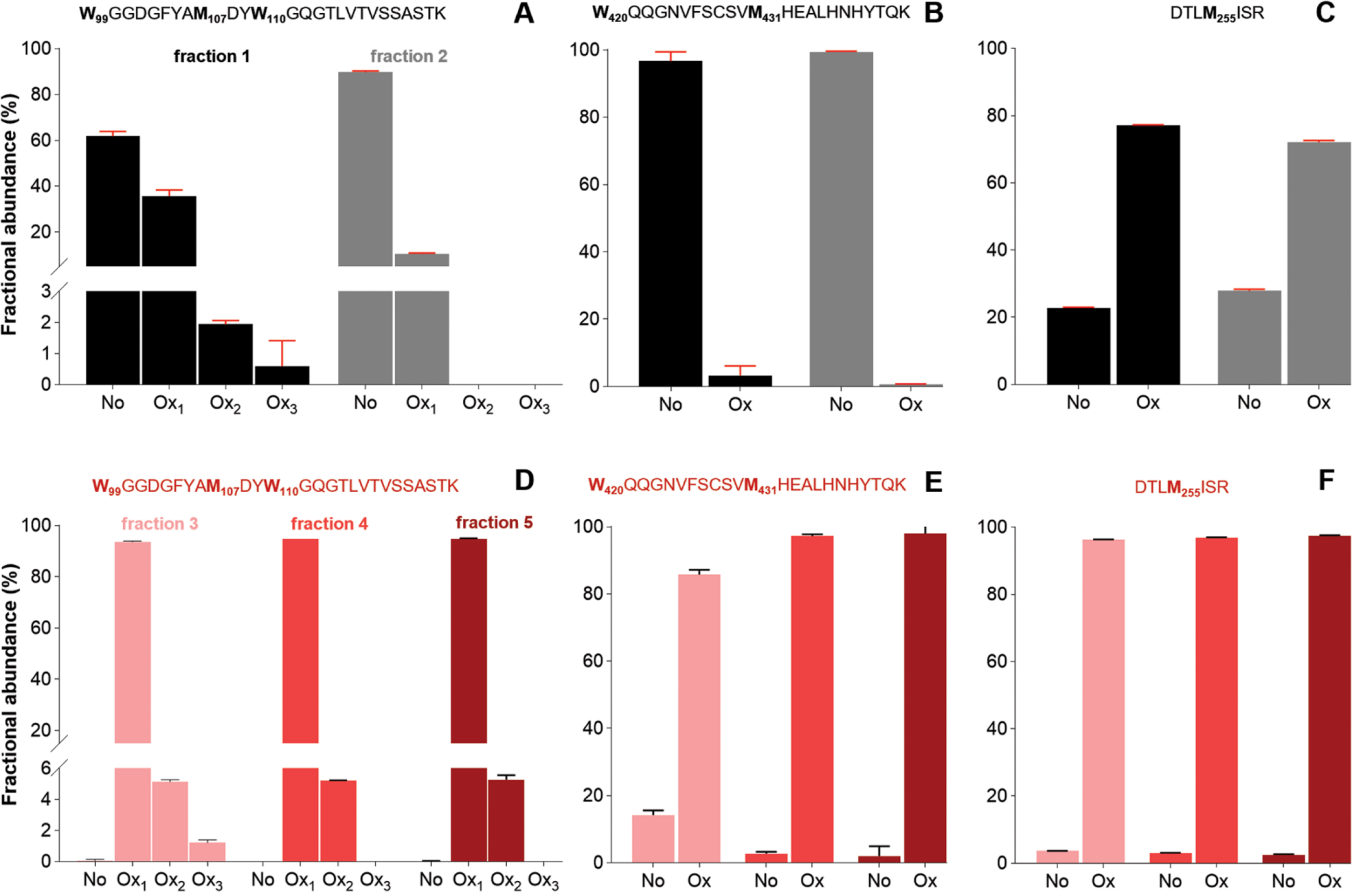
Relative abundance of oxidized peptides in the trastuzumab reference standard (A–C) for fractions 1 and 2, and in trastuzumab after forced oxidation (D–F) for fractions 3, 4, and 5, as determined with LC–MS/MS peptide mapping.

No significant difference in *N*-glycosylation pattern was observed between the pre- and main peak in the trastuzumab reference sample (Figure S1), indicating that different glycan structures are not contributing to a separation of protein variants. Figure 4 displays the fractional abundance of oxidized peptides in the reference sample (Figure 4A–C) and in trastuzumab after forced oxidation (Figure 4D–F). Note that abundance ratios (e.g., no oxidation vs oxidation) can be directly compared between peptides, but absolute quantitation between different fractions does not apply. The fractional abundance of oxidation and deamidation variants exhibited variations between the pre- and main peaks of the reference sample, and these variations were specific to each peptide. For instance, in the case of the **W**_**420**_QQGNVFSCSV**M**_**431**_HEALHNHYTQK peptide, the fractional abundance of oxidation in the prepeak and main peak was determined to be 3 and 1%, respectively. For the DTL**M**_**255**_ISR peptide, the fractional abundances of oxidation were found to be 77 and 72%, respectively. In both cases, no appreciable difference in oxidation abundance was observed between the prepeak and main peaks that resulted in major retention time shifts. For **W**_**99**_GGDGFYA**M**_**107**_DY**W**_**110**_GQGTLVTVSSASTK, the fractional abundance of oxidation in the prepeak was 36% for singly oxidized peptides, and a slightly lower value (23%) was determined for the prepeak (before forced oxidation). This peptide is found in the CDR loop and has been reported as an oxidation hotspot in trastuzumab due to its surface exposure.^6^ Oxidation of surface-exposed residues, potentially originating from environmental or downstream processes, will result in the increased formation of hydrophilic variants eluting prior to the main variant. The prepeak is enriched with hydrophilic variants, hence eluting prior to the main peak.

For IYPT**N**_**55**_GYTR and VS**N**_**328**_K, the fractional deamidation abundance was determined to be 2 and 73%, respectively, but again, no significant difference between the pre- and main peaks was found that contributes to shifts in retention time. In contrast, for the light-chain peptide ASQDV**N**_**30**_TAVAWYQQKPGK, the fractional abundance of deamidated asparagine in the main peak was 1%, whereas the prepeak reached 45% (see Figure S2), which may largely explain the retention time shift displayed in Figure 3. The elevated level of deamidation could potentially be originating from upstream processing and cell culture or downstream processing and storage.^27^

Next, the oxidation levels between the reference and trastuzumab samples after forced oxidation were compared. No oxidation was detected for many of the peptides harboring tryptophan residues (see peptides in Table S3), suggesting that these residues are not surface exposed. Upon forced oxidation, the ratios of oxidized species changed drastically for the peptides displayed in Figure 4D–F, yielding ≫86% fractional oxidative abundance. The CDR peptide, **W**_**99**_GGDGFYA**M**_**107**_DY**W**_**110**_GQGTLVTVSSASTK, showed complete oxidation after exposure to the oxidant with >80% oxidation in all fractions for the single-oxidized form. The double oxidized form was generally low, <6% in all fractions, and triple oxidation was observed only in fraction 1, although at a very low level. Exposed tryptophan residues are also known to increase the propensity for aggregation. Aggregation results in enriched level of hydrophobic variants, which partly explains the shift in retention.^28^ DTL**M**_**255**_ISR and **W**_**420**_QQGNVFSCSV**M**_**431**_HEALHNHYTQK in the Fc region of trastuzumab is situated close to the CH2–CH3 interface, potentially resulting in changed effector functions or reduced binding to protein A. Oxidation of these residues has been reported to activate the unfolding of IgG,^29,30^ hence increasing the accessible surface area. The CH2–CH3 domains are also less stable and known to trigger conformational change.^31^ This contributes to a larger retention time shift in comparison with the main peak in the reference sample. Interestingly, for ASQDV**N**_**30**_TAVAWYQQKPGK, it was observed that there was a variation in fractional deamidation abundance in the stressed sample, with peak 3 reaching 15% deamidation, peak 4 reaching 5%, and peak 5 reaching 4%. This will also in part contribute to the retention time shifts, where retention time appears to decrease with an increased deamidation level.

## Conclusions

We highlight the critical role of phase systems for tuning chromatographic retention and selectivity. To effectively profile molecular mAb variants, a stationary phase chemistry offering complementary interaction modes, such as van der Waals interactions, H-bonding, π–π interactions, and steric interactions, presents abundant opportunities for unparalleled selectivity. In addition, protein–salt interactions can play a key role in achieving high-resolution HIC separation. While (NH_4_)_2_SO_4_ is widely regarded as the gold standard for HIC, chloride ions have been identified as a crucial factor in achieving selectivity, leading to the resolution between distinct molecular antibody variants. Although the linear solvent strength (LSS) plots for mAbs are relatively steep; retention in HIC is not based on an on–off mechanism. Consequently, the increasing column length has a profoundly positive effect on the resolution. This also indicates that the chromatographic performance can likely be further optimized when downscaling the particle diameter and increasing the operating pressure while ensuring protein conformation is not affected. Nonetheless, selectivity mediated by the ligand architecture and salt type should be prioritized during mAb variant analysis. Finally, our study also demonstrated that the site-specific deamidation of trastuzumab lowers the retention time. However, deamidated species can only be separated from the nondeamidated fraction in the presence of chloride ions. Also, site-specific oxidation induces protein unfolding, which, in turn, increases the retention time due to increased hydrophobic residue exposure.

## Supporting Information Available

Description of the equations to determine the retention equilibration constant and lists the values established for *S* and *k*′_b_ in Table S2, an overview of glycovariants present in the trastuzumab reference material (Figure S1), and a list of post-translational modifications in the trastuzumab reference sample and trastuzumab after forced oxidation (Table S3) (PDF)
