# Supplementary material for: Selectivity and Resolving Power of Hydrophobic Interaction Chromatography Targeting the Separation of Monoclonal Antibody Variants
Source: Anal Chem. 2024 Jan 8;96(3):1121–8. doi: 10.1021/acs.analchem.3c04011 (PMC10809212; doi:10.1021/acs.analchem.3c04011)
Supplement: Supplementary file 1 — ac3c04011_si_001.pdf [file ac3c04011_si_001.pdf]

Supporting Information:

Selectivity and resolving power of hydrophobic interaction chromatography targeting the separation of monoclonal antibody variants

Raphael Ewonde Ewonde[1], Katharina Böttinger[2], Jelle De Vos[1,†], Nico Lingg[3], Alois Jungbauer[3], Christopher A. Pohl[4], Christian G. Huber[2], Gert Desmet[1], Sebastiaan Eeltink[1*]

[1]Vrije Universiteit Brussel (VUB), Department of Chemical Engineering, Pleinlaan 2, 1050, Brussels, Belgium

[2]University of Salzburg, Department of Biosciences and Medical Biology, Bioanalytical Research Labs Hellbrunner Strasse 34, 5020 Salzburg, Austria

[3]University of Natural Resources and Life Sciences, Institute of Bioprocess Science and Engineering, Department of Biotechnology, Muthgasse 18, 1190 Vienna, Austria

[4]CAP Chromatography Consulting, Sunnyvale, 94085, California, USA

(*) Corresponding author

Pleinlaan 2, B-1050, Brussels, Belgium

Tel.: +32 (0)2 629 3324, Fax: +32 (0)2 629 3248, E-mail: Sebastiaan.Eeltink@vub.be

† Current affiliation is: RIC group, President Kennedypark 26, B-8500 Kortrijk, Belgium

Table of Content

1. Estimating the Retention Equilibrium Constant (*K*) S-2

2. Determination of Retention Parameters S-3

3. RP-HPLC-MS/MS Peptide Profiling of Major HIC Fractions S-4

4. References S-6

1. Estimating the Retention Equilibrium Constant *(K)*

To compare the hydrophobicity of the different columns, we define here the retention equilibrium constant, K. Adopting an adsorption-based retention mechanism and assuming the analyte retention is proportional to the surface area available for retention, we can write:

$$k' = (S'/V_m) \cdot K \qquad (1)$$

where k' is the retention factor of the analyte, S' the area of the surface upon which retention is occurring, V_m the volume of the mobile phase, and K is the corresponding equilibrium constant given by:

$$K = (n/S')_e/C_{m,e} \qquad (2)$$

where n is number of moles in equilibrium with the concentration in the mobile phase and $C_{m,e}$ is equilibrium mobile phase concentration. The subscript "*e*" in each term represents equilibrium. K can be used as an index for hydrophobicity as it defines the number of moles of the analyte retained on the surface area S' in equilibrium with the mobile-phase concentration. The higher its value the more hydrophobic the stationary phase.

To obtain K, we need to determine the k' of an analyte and the S'/V_m ratio of the stationary phase. The k' for phenanthrene was measured in isocratic mode for each column applying a mobile phase of 30:70 (v/v)% acetonitrile: 0.1M ammonium acetate. The column hold-up time (t_0) was determined by injecting a water plug. The column was operated at 1 mL/min and the column was thermostatted at 30°C. The S'/V_m ratio can be derived from the relationship mass (m) = density (ρ) × volume (V). For the porous alkylamide and polyalkylimide particles, the S' values were determined by the manufacturer to be 20 m^2/g, which corresponds to $20 \cdot 10^{-3}\ m^2/kg_{silica} \cdot m_{silica}$. Consequently,

$$m_{silica} = \rho_{silica} * V_p\,(1-\varepsilon_p) = \rho_{silica} * V_c\,(1-\varepsilon_e)(1-\varepsilon_p) \qquad (3)$$

where the term $V_p\,(1-\varepsilon_p)$ represents the volume of the silica particles excluding the mesopores, V_p is the volume of the particles which is equivalent to $V_c\,(1-\varepsilon_e)$, V_c is the column volume, ε_p is the porosity inside the particle (~30%), and ε_e is the external porosity (~40%). Porosity values have been obtained Cabooter *et al.*[1] Considering the density of silica (ρ_{silica} = $2.2 \cdot 10^3$ kg/m^3), S' becomes:

$$S' = (20 \cdot 10^{-3}\ m^2/kg_{silica}) \cdot (2.2 \cdot 10^3\ kg/m^3) \cdot [V_c\,(1-\varepsilon_e)\cdot(1-\varepsilon_p)] = 44 \cdot V_c\,(1-\varepsilon_e)\cdot(1-\varepsilon_p) \qquad (4)$$

The volume of the mobile phase (V_m) can be expressed as:

$$V_m = V_c\,(\varepsilon_e + (1-\varepsilon_e)\cdot\varepsilon_p) \qquad (5)$$

where $(\varepsilon_e + (1-\varepsilon_e)\cdot\varepsilon_p)$ corresponds to the total porosity (ε_T). Therefore, the S'/V_m ratio becomes:

$$\frac{S'}{V_m} = \frac{44 \cdot (1-\varepsilon_e)(1-\varepsilon_p)}{(\varepsilon_e + (1-\varepsilon_e) \cdot \varepsilon_p)} \quad (6)$$

For the nonporous particle (butyl), $\varepsilon_p = 0$, and the ratio S'/V_m is estimated by:

$$\frac{S'}{V_m} \sim \frac{6 \cdot (1-\varepsilon_e)}{\varepsilon_e} \quad (7)$$

By substituting the values of ε_p and ε_e in Eq. (6) and (7) and then S'/V_m and k' in Eq. (1), the estimated values of K were obtained. Values have been reported in Table S1.

Table S1. Estimated retention equilibrium constant (K) for the different HIC stationary phases.

Chemistry	Pore size (Å)	t_0 (min)	S'/V_m	k'	K
Butyl	/	0.83	9.00	17.67	1.963
Alkyl amide	1000	1.50	31.86	1.842	0.058
Polyalkyl amide	1000	1.50	31.86	0.221	0.007

2. Determination of Retention Parameters

The logarithmic retention factor of analytes in HIC can be approximated by the following linear relation based on the LSS model mode:

$$\ln k' = -\ln k'_b + S \cdot [M] \quad (4)$$

where $[M]$ is the molar salt concentration, $\ln k'_b$ the extrapolated value of $\ln k'$ to aqueous buffer solution. The $\ln k'_b$ (intercept) and S (slope) values listed in Table S2 were determined by injecting trastuzumab reference sample on to the different columns (butyl, alkylamide, polyalkylimide) while operating the columns in isocratic mode and applying $(NH_4)_2SO_4$ or NaCl as modifiers, respectively.

Table S2. k'_b and S data determined for the trastuzumab reference sample.

**Column**	**Salt**	***ln k'b***	***S***	***R^2***
Butyl	$(NH_4)_2SO_4$	18.69	8.33	0.9886
Alkyl amide	$(NH_4)_2SO_4$	11.70	4.65	0.9976
Polyalkyl amide	$(NH_4)_2SO_4$	4.04	2.31	0.9979
Butyl	NaCl	25.90	35.97	0.9974
Alkyl amide	NaCl	14.05	16.91	0.9994
Polyalkyl amide	NaCl	5.01	9.5	0.9984

3. RP-HPLC-MS/MS Peptide Profiling of Major HIC Fractions

Initially the glycosylation patterns of fraction 1 (pre peak) and 2 (main peak), in trastuzumab reference sample, were compared, considering the glycopeptide, EEQY**N**$_{297}$STYR, which carries this oligosaccharide, see Fig. S1. The standard deviations are low, and the glycosylation pattern is homogenous between both fractions. The dominant glycoforms are of complex type with one or two terminal N-acetylglucosamine (A), zero terminal galactose (G) and core-fucose (F); A2G0F and A1G0F, accounting for approximately 57% and 20% fractional abundance, respectively. The remaining glycoforms were below 10%.

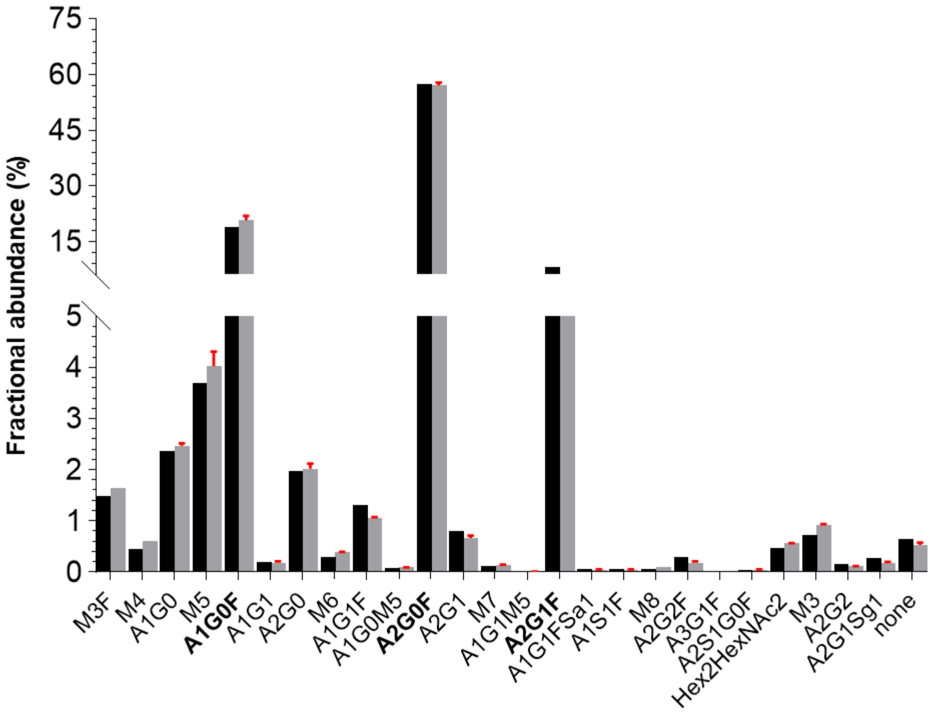

Figure S1. Comparison of glycovariants in fractions 1 (black) and 2 (grey) from trastuzumab reference material. LC-MS conditions: peptides were separated on a 75 µm i.d. × 150 mm column packed with 2 µm C_{18} particles (100 Å) utilizing a two-step linear gradient from 1-30% in 35 min and then 30-60% in 10 min (with 0.1% formic acid in acetonitrile). The column was operated at a flow rate of 300 nL min^{-1} applying 50°C. MS conditions are described in the 'Experimental Section' in the main manuscript.

In the next step, all possible tryptic peptides of trastuzumab with potential modification sites were screened. The protein sequence of trastuzumab from DrugBank (Accession number: DB00072) is presented below in FASTA format and peptides with modifications potentially are listed in Table S3. Some peptides contained more than one modification site, all of which were considered in the analysis.

>Anti-HER2 Light chain (1 and 2)

DIQMTQSPSSLSASVGDRVTITCRASQDVNTAVAWYQQKPGKAPKLLIYSASFLYSGVPSRFS
GSRSGTDFTLTISSLQPEDFATYYCQQHYTTPPTFGQGTKVEIKRTVAAPSVFIFPPSDEQLKSG
TASVVCLLNNFYPREAKVQWKVDNALQSGNSQESVTEQDSKDSTYSLSSTLTLSKADYEKH
KVYACEVTHQGLSSPVTKSFNRGEC

>Anti-HER2 Heavy chain (1 and 2)

EVQLVESGGGLVQPGGSLRLSCAASGFNIKDTYIHWVRQAPGKGLEWVARIYPTNGYTRYA
DSVKGRFTISADTSKNTAYLQMNSLRAEDTAVYYCSRWGGDGFYAMDYWGQGTLVTVSSA
STKGPSVFPLAPSSKSTSGGTAALGCLVKDYFPEPVTVSWNSGALTSGVHTFPAVLQSSGLYS
LSSVVTVPSSSLGTQTYICNVNHKPSNTKVDKKVEPKSCDKTHTCPPCPAPELLGGPSVFLFPP
KPKDTLMISRTPEVTCVVVDVSHEDPEVKFNWYVDGVEVHNAKTKPREEQYNSTYRVVSVL
TVLHQDWLNGKEYKCKVSNKALPAPIEKTISKAKGQPREPQVYTLPPSREEMTKNQVSLTCL
VKGFYPSDIAVEWESNGQPENNYKTTPPVLDSDGSFFLYSKLTVDKSRWQQGNVFSCSVMH
EALHNHYTQKSLSLSPGK

Table S3. Screening of post-translational modifications in trastuzumab reference sample and trastuzumab after forced oxidation.

**Peptides** ([a])	**Subunit**	**PTM** ([b])
NTAYLQ**M**$_{83}$NSLR	C_H	Ox.
**W**$_{99}$GGDGFYA**M**$_{107}$DY**W**$_{110}$GQGTLVTVSSASTK	C_H	Ox.
DTL**M**$_{255}$ISR	C_H	Ox.
FN**W**$_{280}$YVDGVEVHNAK	C_H	Ox.
VVSVLTVLHQD**W**$_{316}$LNGK	C_H	Ox.
**W**$_{420}$QQGNVFSCSV**M**$_{431}$HEALHNHYTQK	C_H	Ox.
DIQ**M**$_{4}$TQSPSSLSASVGDR	C_L	Ox.
ASQDVNTAVA**W**$_{35}$YQQKPGK	C_L	Ox.
**E**$_{1}$VQLVESGGGLVQPGGSLR	C_H	pE
ASQDV**N**$_{30}$TAVAWYQQKPGK	C_L	Deam.
IYPT**N**$_{55}$GYTR	C_H	Deam.
VS**N**$_{328}$K	C_H	Deam.
**Peptide** ([a,c])		
DTYIH**W**$_{36}$VR	C_H	Ox.
GLE**W**$_{47}$VAR	C_H	Ox.
DYFPEPVTVS**W**$_{161}$NSGALTSGVHTFPAVLQSSGLYSLSSVVTVPSSSLG TQTYICNVNHKPSNTK	C_H	Ox.

GFYPSDIAVE**W**$_{384}$ESNGQPENNYK	C_H	Ox.
VQ**W**$_{148}$K	C_L	Ox.

([a]) The different peptides considered in this study with potential modification sites highlighted in bold and the position of the amino acid written as subscript.

([b]) the following post-translational modifications were considered for quantification: oxidation (ox.), pyroglutamate formation (pE), and deamidation (deam.).

([c]) No oxidation was detected on the tryptophane residues in these peptides which corresponds to 100% fractional abundance (unoxidized) in all fractions.

Trastuzumab contains the PENNY-motif in the Fc subdomain, which is susceptible to deamidation.[2,3] However, we did not observe deamidation at these positions GFYPSDIAVEWES**N**$_{387}$GQPE**N**$_{392}$**N**$_{393}$YK in any of the fractions. For the light-chain peptide ASQDVN30TAVAWYQQKPGK, the fractional abundance of deamidated asparagine in the main peak was 1%, whereas the pre-peak reached 45%, see Fig. S2.

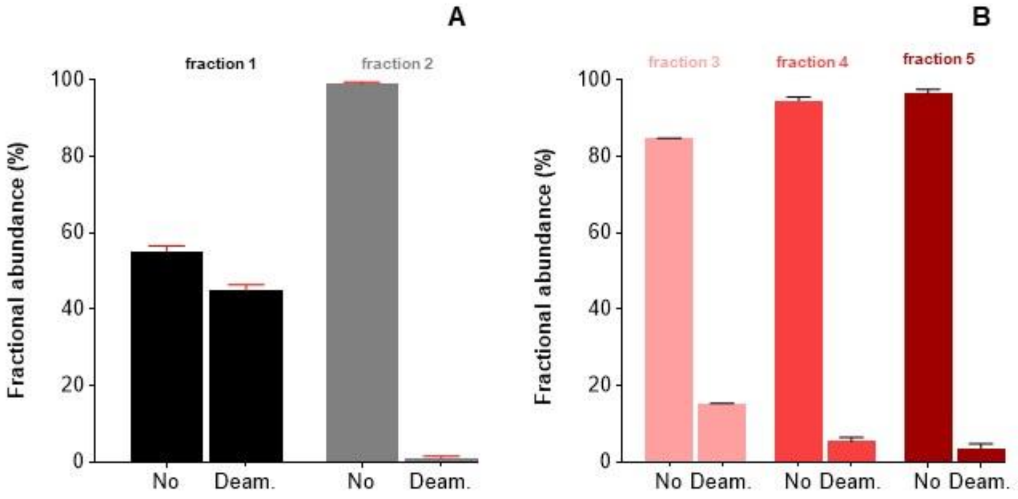

Figure S2. Deamidation level of peptide ASQDV**N**$_{30}$TAVAWYQQKPGK in trastuzumab reference material **(A)** and trastuzumab after oxidation **(B)**.

4. References

(1) Cabooter, D.; Lynen, F.; Sandra, P.; Desmet, G. Total Pore Blocking as an Alternative Method for the On-Column Determination of the External Porosity of Packed and Monolithic Reversed-Phase Columns. *J. Chromatogr. A* **2007**, *1157* (1–2), 131–141.

(2) Di Marco, F.; Berger, T.; Esser-skala, W.; Rapp, E.; Regl, C.; Huber, C. G. Simultaneous Monitoring of Monoclonal Antibody Variants by Strong Cation-exchange Chromatography Hyphenated to Mass Spectrometry to Assess Quality Attributes of Rituximab-based Biotherapeutics. *Int. J. Mol. Sci.* **2021**, *22* (16).

(3) Alam, M. E.; Barnett, G. V.; Slaney, T. R.; Starr, C. G.; Das, T. K.; Tessier, P. M. Deamidation Can Compromise Antibody Colloidal Stability and Enhance Aggregation in a pH-Dependent Manner. *Mol. Pharm.* **2019**, *16* (5), 1939–1949.
